# Cytotoxic activities of selected medicinal plants from Iran and phytochemical evaluation of the most potent extract

**Published:** 2009

**Authors:** S. Sahranavard, F. Naghibi, M. Mosaddegh, S. Esmaeili, P. Sarkhail, M. Taghvaei, S. Ghafari

**Affiliations:** 1 *Department of Pharmacognosy, School of Pharmacy, Shaheed Beheshti University of Medical Sciences, Tehran, I.R.Iran*; 2 Traditional Medicine & Materia Medica Research Center, Shaheed Beheshti University of Medical Sciences, Tehran, I.R.Iran

**Keywords:** Cytotoxicity, **Ferula szowitsiana**, Monoterpenoid

## Abstract

Methanolic extract of 15 Iranian medicinal plants were prepared and tested for their cytotoxic activities against three cancer cell lines (MCF7, HepG2, WEHI164) and one normal cell line (MDBK). Some plants showed cytotoxic activities. The extract of *Ferula szowitsiana* root, which proved to be the most active, was chosen for further phytochemical studies. The major compounds of the most potent acetone extract were isolated. They were identified as chimgin and chimganin, two known monoterpenoids, by spectroscopic means. Their cytotoxic activity was evaluated in three cell lines. The results show that these compounds are responsible, at least in part, for the cytotoxic activity of this plant.

## INTRODUCTION

Cancer is a general term applied to a series of malignant diseases which may affect many different parts of the body. If the process is not arrested, it may progress until it causes the death of the organism(1). Cancer is one of the major causes of death in developed countries, together with cardiac and cerebrovascular diseases(2). Conventional cancer treatments include surgery, radiation and chemotherapy. The dispersed nature of end-stage disease drives the need for systematic therapy and chemotherapy aims to wipe out all cancerous colonies within the patient’s body, including metastasized cancer cells(3). Currently, much commonly used anticancer therapeutics represent broadly cytotoxic agents. These agents have been frequently discovered using cell-based cytotoxicity assays.

Drug discovery from medicinal plants has played an important role in the treatment of cancer and, indeed, most new clinical applications of plant secondary metabolites and their derivatives over the last half century have been applied toward combating cancer(4,5).

Traditional medicine over the years has proved to be an invaluable guide in drug discovery and Iran has a long history in this field and is a great source of new bioactive compounds.(6,7).

In this study we collected 15 plant species from different parts of Iran. These plants have ethnomedicinal reports or have shown antifungal properties in a previous screening of native Iranian plants for their *in vitro* antifungal activity against 19 fungal strains(8,9). The methanolic extracts of these plants were prepared and their antiproliferative activities were screened against different cancer and normal cell lines, i.e. MCF7 (human breast carcinoma), HepG2 (hepatocellular carcinoma), WEHI (fibrosarcoma) and MDBK (cow’s normal kidney cell). Extract of *F. szowitsiana* showed the most potent antiproliferative activity against human tumor cells. This plant is used in folk medicine for the treatment of various diseases such as dermal wounds and asthma(6). Fractionation of the methanolic extract with acetone led to the isolation of two cytotoxic compounds. Both these compounds have previously been isolated from some Ferula species but this is the first report of their isolation from *F. szowitsiana* (10–12).

## MATERIALS AND METHODS

All solvents were purchased from Merck, Germany. NMR spectra were recorded on a Bruker Ultra shild NMR spectrometer (500 MHz for ^1^Hand 125 MHz for ^13^CNMR) using CDCl_3_. Electron Impact mass spectra were obtained using a Finnigan MAT-EI-TSQ at 70 eV. Melting points were determined on a Reichert-jung apparatus and UV spectra were taken on a Shimadzu UV-3100 spectro-photometer.

### 

#### Plants and extracts

The selected plants (Table 1) were collected at different localities of Iran and were identified at Traditional Medicine & Materia Medica Research Center, Shaheed Beheshti University of Medical Sciences, Tehran, Iran. Voucher specimens of the plants were deposited in the herbarium of the Traditional Medicine and Materia Medica Research Center. The shade dried and ground plant parts were extracted with methanol for 24 h while stirring at room temperature. The solvent was evaporated under reduced pressure at a temperature of 45°C. The resulting crude extracts were stored until assayed.

**Table 1:** Plant species investigated in this study

Plant name	Family	Part used	Traditional uses	Voucher
*Anchusa azurea Mill.*	Boraginaceae	Aerial part	Cold, sedative	TMRC 273
*Biebersteinia multifida* DC.	Geraniaceae	Root	Dermal wounds	TMRC 486
*Buxus hyrcana* Pojark.	Buxaceae	Aerial part	Antifungal	TMRC 1161
*Caccinia macranthera* (Branks & soland.) Brand.	Boraginaceae	Root	Dermal infections, liver disorders, dyspepsia	TMRC 510
*Capparis spinosa* L.	Capparidaceae	Aerial part	Rheumatism, headache, digestive disorders, hemorrhoid	TMRC 1295
*Chenopodium butrys* L	Chenopodiaceae	Aerial part	Antifungal	TMRC 1296
*Convolvulus commutatus* Boiss.	Convolvulaceae	Aerial part	Infected wounds	TMRC 564
*Echium italicum* L.	Boraginaceae	Aerial part	Dermal wounds	TMRC 1299
*Ferula szowitsiana* DC.	Apiaceae	Root	Dermal wound, asthma, cough	TMRC 965
*Glaucium oxylobum* Boiss.& Buhse	Papaveraceae	Aerial part	Antifungal	TMRC 1283
*Leontice leontopetalum* L.	Berberidaceae	Root	Rheumatism, joint pain and inflammation	TMRC 1287
*Parrotia persica* (DC.) C. A. Mey	Hammamelidaceae	Bark	Broken bone	TMRC 1281
*Perovskia abrotanoides* Karel.	Lamiaceae	Root	Leishmaniasis	TMRC 801
*Stachys turcomanica* Trautv.	Lamiaceae	Aerial part	Influenza, bronchitis, footinflammation, toothache	TMRC 491
*Zygophyllum fabago L.*	Zygophylaceae	Aerial part	Digestive problems, Diarrhea	TMRC 2189

#### Cytotoxicity assay

The cytotoxicity bioassay was performed against three cancer cell lines (MCF-7, HepG2, WEHI) and one normal cell line (MDBK). Cells were grown in monolayer cultures in Dulbecco’s Modified Eagle’s Medium (DMEM) and RPMI 1640 supplemented with 5% FBS (Gibco), 100 U/ml penicillin, 10 µg/ml streptomycin, and maintained at 37°C in a 5% CO_2_ incubator. For testing, cells were washed with PBS (phosphate buffer saline), harvested by tripsinization, plated (10^4^ cell/well) in 96-well plates, and incubated for 24 h at 37°C in the incubator. Afterwards, they were exposed to different concentrations of plant extracts and incubated for further 72h followed by MTT [3-(4,5- dimethylthiazol-2yl)-2,5- biphenyl tetrazolium bromide] assay at 570 nm(13). Viability was defined as the ratio (expressed as a percentage) of absorbance of treated cells to untreated cells. The selectivity index (SI) was determined as the ratio of the concentration at which growth was inhibited by 50% (IC_50_) on normal cells to cancer cells.

#### Extraction and isolation

The ground roots of *F. szowitsiana* (40 g) which were collected from the Kalaleh region in Golestan province, at the height of 500-700 m, were extracted with methanol over night. After filtration, the solvent was evaporated to dryness under reduced pressure at 40°C. The residue was extracted by acetone. The dried acetone extract (6 g, semisolid reddish brown gum) was fractioned by column chromatography using Silica gel 60 eluting first with hexane, followed by a gradient of hexane CHCl_3_ up to 100% CHCl_3_ and CHCl_3_ -acetone up to 15% acetone. Eight fractions were obtained. Fraction 3 was separated by Preparative Thin Layer Chromatography (PTLC) over silica gel, using CH_2_ Cl_2_ : EtOAc (9.5:0.5) as mobile phase to obtain 175 mg compound 1 (Chimganin). Fraction 5 was crystallized from EtOH twice and afforded 500 mg of compound 2 (Chimgin).

#### Chimganin (1,7,7-Trimethylbicyclo[2.2.1] heptan-2-yl-4-hydroxy-3-methoxybenzoate) (compound 1)

Colorless crystals, m.p.80-82 °C (lit. 85 °C);^1^ HNMR (500 MHz, CDCl_3_) (α=endo, β=exo): δ 7.70 (1H, dd, H-6’), 7.60 (1H, d, H-2’), 6.98 (1H, d, H-5’), 5.12 (1H, dt, H-2β), 3.96 (3H, s, OMe), 2.50 (1H, m, H-3β), 1.16 (1H, m, H-3α), 2.16 (1H, m, H-6β), 1.45 (1H, m, H-6α), 1.84 (1H, m, H-5β), 1.34 (1H, m, H-5α), 1.76 (1H, t, H-4), 1.13 (3H, s, H-9), 0.95 (3H, s, H-8), 0.94 (3H, s, H-10).^13^ C NMR (125 MHz, CDCl_3_): 167.13 (C-7’),150.28 (C-4’), 146.59 (C-3’), 124.36 (C-6’), 114.51 (C-5’), 123.40 (C-1’), 112.23 (C-2’), 80.75 (C-2), 56.46 (OMe), 49.50 (C-7), 48.27 (C-1), 45.42 (C-4), 37.34 (C-3), 28.52 (C-5), 27.54 (C-6), 19.92 (C-8), 19.34 (C-9), 14.05 (C-10). EIMS (70 eV) m/z (rel.int.): 304 (M^+^) (15), 136 (10), 121 (100), 118 (35), 93 (20).

#### Chimgin (1,7,7- Trimethylbicyclo [2.2.1] heptan-2-phydroxybenzoate) (compound 2)

White needles, m.p. 150-152 °C (lit. 154-155 °C).

^1^ H NMR (500 MHz, CDCl_3_) (α=endo, β=exo):δ 7.92 (1H, d, H-6’), 7.92 (1H, d, H-2’), 6.88 (1H, d, H-5’), 6.88 (1H, d, H-3’), 5.05 (1H, dt, H-2β), 2.45 (1H, m, H-3β), 1.09 (1H, dd, H-3α), 2.13 (1H, m, H-6β), 1.40 (1H, m, H-6α), 1.77 (1H, m, H-5β), 1.31 (1H, m, H-5α), 1.71 (1H, t, H-4), 0.95 (3H, s, H-9), 0.90 (3H, s, H-10), 0.89 (3H, s, H-8).^13^ CNMR (125 MHz, CDCl_3_): 167.34 (C-7’), 161.99 (C-4’), 132.01 (C-6’), 132.01 (C-2’), 115.73 (C-3’), 115.73 (C-5’), 122.44 (C-1’), 80.38 (C-2), 48.22 (C-1), 49.44 (C-7), 45.41 (C-4), 37.33 (C-3), 28.49 (C-5), 27.79 (C-6), 20.12 (C-8), 19.30 (C-9), 13.10 (C-10). EIMS (70 eV) *m/z* (rel.int): 274(M^+^) (15), 153 (10) 136 (37), 121 (100), 93 (50).

#### Cytotoxicity determination of Chimgin and Chimganin

The cytotoxicity of two isolated compounds was evaluated against MCF-7, HepG2 and MDBK cell lines by the MTT assay.

## RESULTS

The cytotoxic activity of 15 different medicinal plants of Iran against MCF-7, HepG2, WEHI and MDBK cell lines was evaluated. The results of this screening are summarized in Table 2. While most of the extracts appeared almost inactive (IC50 values above 100 µg/ml)(14), the methanolic extract of *F. szowitsiana* (yield 22.4%) showed a promising antiproliferative activity. It showed IC50 values less than 100 µg/ml in all evaluated cell lines and in 3 cell lines out of 4 it showed the highest cytotoxicity. Further-more, *Perovskia abrotanoides* exhibited moderate cytotoxicity against three cell lines, whereas *Buxus hyrcana* and *Parrotia persica* where only active against one cell line. According to the results of the bioassay, *F. szowitsiana* was selected for further studies in order to identify the active compounds.

**Table 2:** *In vitro* cytotoxicity of methanol extracts of selected medicinal plants. Tamoxifen was used as positive control

Plant name	IC50 a value			
	MCF-7	HepG2	WEHI	MDBK
*Anchusa azurea Mill.*	>100	>100	>100	>100
*Biebersteinia multifida* DC.	>100	>100	>100	>100
*Buxus hyrcana* Pojark.	44.4	>100	>100	>100
*Caccinia macranthera* (Branks & soland.)	>100	>100	>100	>100
*Capparis spinosa* L.	>100	>100	>100	>100
*Chenopodium butrys L*	>100	>100	>100	>100
*Convolvulus commutatus* Boiss.	>100	>100	>100	>100
*Echium italicum* L.	>100	>100	>100	>100
*Ferula szowitsiana* DC.	29	40.6	79	38
*Glaucium oxylobum* Boiss.& Buhse	>100	>100	>100	>100
*Leontice leontopetalum* L.	>100	>100	>100	>100
*Parrotia persica* (DC.) C. A. Mey	>100	>100	97.4	>100
*Perovskia abrotanoides* Karel.	93	>100	40.6	62.3
*Stachys turcomanica* Trautv.	>100	>100	>100	>100
*Zygophyllum fabago L.*	>100	>100	>100	>100
Tamoxifen	6.5	13.8	89.6	19.1

a The concentration at which growth was inhibited by 50%, µg/ml

**Fig. 1:**
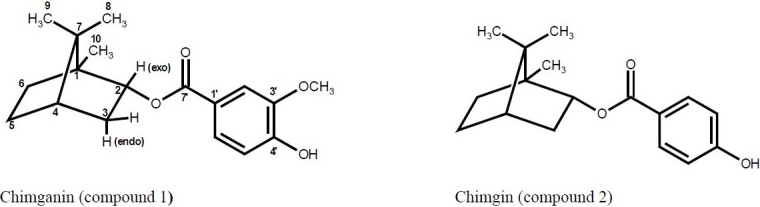
Structures of the isolated monoterpenoids

The bioassay-guided fractionation of the methanolic extract led to isolation of two monoterpenoids (Fig. 1). The ^1^H NMR of compound 1 revealed the occurrence of a 1,2,4- trisubstituted aromatic system. In addition it showed characteristic signals of a bornyl moiety in which H-2 (exo) appears as a doublet of triplets (J = 9.5, 2.9 Hz) due to a “W” coupling to the coplanar H-6 (exo)(15). By comparison of its spectroscopic data with the literature values(16), it was identified as chimganin. Compound 2 showed similar NMR data but was characterized by a p-substituted aromatic system and identified as chimgin(16). The cytotoxicity of compounds 1 and 2 was evaluated against three different cell lines. IC_50_ was calculated from dose-response curves (Table 3).

**Table 3:** *In vitro* cytotoxicity of isolated compounds

Compounds	IC_50_ (µM)		
	Cell lines		
	MCF-7	HepG2	MDBK
Chimgin (compound 2)	45.2	67.1	69.7
Chimganin (compound 1)	28	74	30.9

## DISCUSSION

This study aimed to investigate the cytotoxic activity of 15 Iranian traditional medicinal plants against three cancer cell lines and one normal cell line. Some plant’s methanolic extracts showed low or no cytotoxicity against the cell lines, whereas *F. szowitsiana* showed the most potent cytotoxicity against all of them.

Bioassay-guided fractionation led to the isolation of two monotepenoids from the root extract of *F. szowitsiana*. Both bornyl esters have been isolated before from different Russian Ferula species(10–12), but are reported here for the first time from *F. szowitsiana*. Previous investigations revealed the occurrence of prenylated coumarin derivatives in the roots of *F. szowitsiana* (17). One of these coumarins, umbelliprenin, inhibited the growth of a human melanoma cell line with an IC_50_ value of 12.3 µM(18).

## CONCLUSION

Both compounds, Chimganin and Chimgin, showed significant cytotoxic effects with lower IC_50_ values compared to the extract and were just slightly less active than tamoxifen which was used as positive control. Thus we conclude that these two compounds are responsible, at least in part, for the observed cytotoxicity of the extract of *F. szowitsiana*.
